# Seaweed carbon removal cannot keep up with climate-driven loss

**DOI:** 10.1371/journal.pbio.3003949

**Published:** 2026-09-08

**Authors:** Karen Filbee-Dexter, Albert Pessarrodona, Kira A. Krumhansl, Thomas Wernberg

**Affiliations:** 1 UWA Oceans Institute & School of Biological Sciences, The University of Western Australia, Perth, Western Australia, Australia; 2 Benthic Communities Group, Institute of Marine Research, Bergen, Norway; 3 Oceans Group, Conservation International, Arlington, Virginia, United States of America; 4 Oceans Group, International Blue Carbon Institute, Singapore, Singapore; 5 Bedford Institute of Oceanography, Fisheries and Oceans Canada, Halifax, Nova Scotia, Canada

## Abstract

The prevailing narrative of seaweed carbon as a possible climate change mitigation solution overlooks the central challenge that climate change itself is adversely impacting seaweed forests worldwide, potentially shifting these ecosystems from carbon sinks to carbon sources. In this Essay, we show that climate-induced seaweed forest loss is eroding their carbon sink capacity three to four orders of magnitude (1,000–10,000×) faster than it can currently be restored or recovered through active interventions. We consider that this stark disparity calls for a fundamental shift in seaweed carbon research away from a focus on future sequestration gains and toward explicitly assessing the risk of carbon emissions from climate-driven ecosystem loss.

## Abstract 2

Seaweed forests are widely viewed as natural climate solutions, yet warming oceans are driving their rapid decline. This Essay reveals that losses are outpacing restoration efforts by orders of magnitude, raising urgent concerns about their role as emerging sources of emissions.

For millennia, our natural ecosystems have absorbed carbon dioxide and stored it in biomass and sediments, helping to regulate Earth’s climate [1]. For example, land forests draw down roughly one third of our carbon dioxide emissions from burning fossil fuels each year [2]. This service has been so reliable that it has become embedded, often implicitly, in climate change projections, mitigation pathways, and solution narratives. Beyond reduction of direct emissions, a commonly suggested ‘solution’ to climate change is to plant trees and restore natural carbon sinks [3]. Yet, emerging evidence suggests that the ability of Earth’s ecosystems to draw down carbon dioxide is weakening [4,5] and its primary producers—the plants and algae—are increasingly affected by accelerating climate change, while internationally-promised global emission reductions to achieve net-zero targets have yet to occur. For forests, climate change can drive wildfires and drought that increases mortality, and warmer temperatures and water limitation can slow growth in some areas, causing some forests to become net emitters of carbon [6]. In Australia, wildfires recently shifted the northeastern rainforest from a carbon sink to a source due to a large tree die-off [7]. In the Amazon, rainforests are drawing down a third less carbon than usual [8]. Arctic permafrost and boreal forests are releasing carbon due to warming and fires [9,10]. As we grapple with this reality on land, similar trends are happening underwater where less visible marine forests are also at risk of transforming from carbon sinks into carbon sources. In the ocean, climate-driven losses of coastal vegetated ecosystems have substantially contributed to land-use change emissions [11].

Seaweed forests are often positioned as marine analogues to terrestrial forests: dynamic, carbon-rich systems that can help offset human emissions if restored and protected at scale [12,13]. This view stems from the high capacity of seaweeds to fix carbon, globally drawing down an estimated 1 billion tonnes of carbon every year through growth [14,15], and the fact that they cover more sunlit coastal area than any other vegetated marine habitat [16]. Seaweeds hold an important role in ocean carbon cycles through the export of organic carbon to deep ocean sinks and sediments, where it can potentially become sequestered for climatically relevant periods [15]. To date, most research on seaweed carbon cycling has focused on understanding the sequestration and carbon dioxide removal (CDR) capacity of seaweed so that it can be harnessed to mitigate climate change [17]. Yet, this emphasis obscures a growing imbalance between the limited scale of interventions to increase seaweed-based CDR (e.g., restoration, afforestation, and farming) and the loss of natural climate benefits (processes that reduce or prevent carbon dioxide emissions) associated with climate-driven declines of seaweed ecosystems.

In this Essay, we show that loss of natural climate benefits resulting from climate-induced seaweed loss may be three to four orders of magnitude (1,000–10,000×) greater than CDR gains achieved through seaweed restoration, afforestation, and farming. We consider that this stark disparity calls for a fundamental shift in seaweed blue carbon research that moves away from a focus on possible sequestration through future interventions gains to an explicit assessment of the extent to which accelerating climate change is reducing the seaweeds natural sequestration capacity, and even leading to net carbon dioxide emissions. At worst, granting offsets for ineffective CDR interventions could enable further emissions when the focus of limited resources should be on protecting and preventing future loss. In the best case, this focus distracts research attention from understanding and addressing the broader challenge: that the capacity of natural systems to absorb carbon is rapidly declining while anthropogenic emissions continue to rise.

## Seaweed forests as blue carbon reservoirs

Seaweeds contribute to carbon sequestration through several pathways, all of which can be affected by climate change and habitat destruction. As they grow, seaweeds absorb carbon through photosynthesis and integrate it into tissue growth. Much of this biomass breaks off as seaweed fragments or floating rafts, which can then leave the high-energy coastal zone [18]. This material can move with ocean currents away from the coast and enter deeper ocean water bodies beyond the continental shelf, where it can be isolated from the atmosphere for decades to centuries [5,15]. Seaweeds also produce and release a large amount of dissolved organic carbon (DOC) into the surrounding seawater, both from living tissue and as detrital seaweed breaks down. A significant portion of this dissolved material is refractory (rDOC), a form of organic carbon that resists biological and environmental degradation and can remain sequestered under in situ environmental conditions for several thousand years [19]. This refractory fraction of seaweed carbon can enter the deep ocean, thereby significantly contributing to ocean carbon sequestration [20,21].

**Fig 1:**
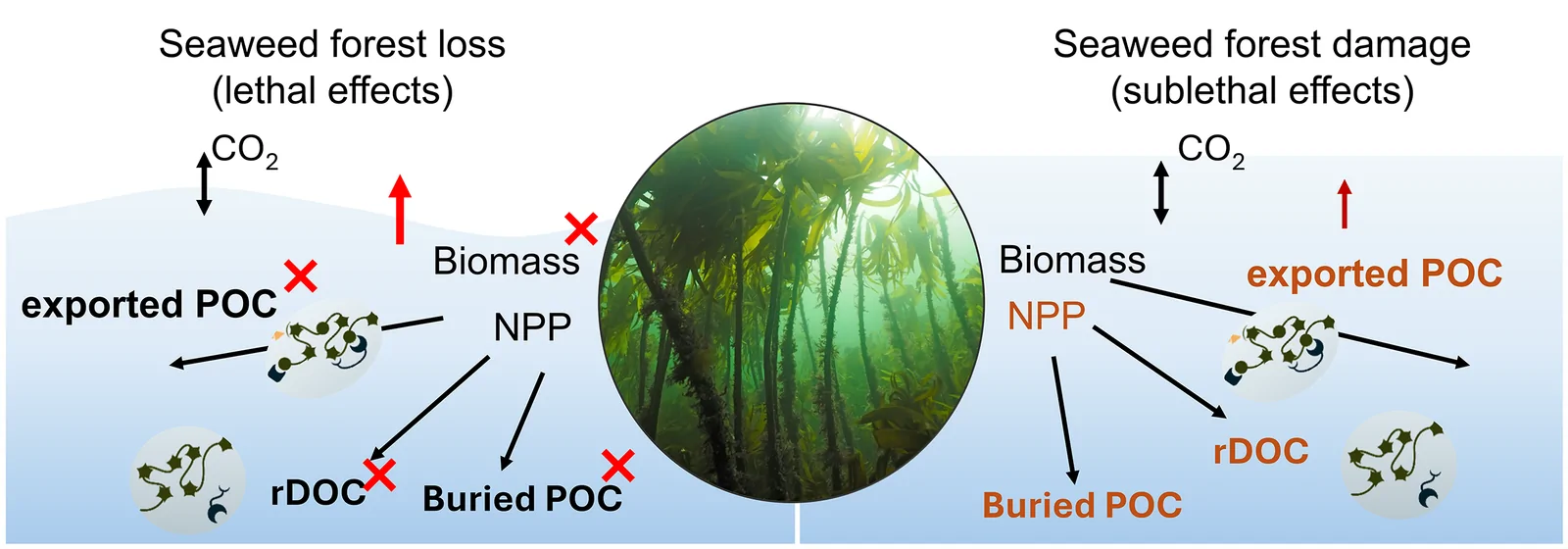
Potential seaweed carbon emission pathways. Illustration showing how seaweed forest loss (lethal effects) and seaweed forest damage (sublethal effects) from warming lead to altered seaweed carbon cycling and translate to marine carbon release. Pathways include changes in standing biomass or stock, net primary productivity (NPP), export of particulate organic carbon (POC) to deep ocean sinks, and release and production of refractory dissolved organic carbon (rDOC). Red crosses represent the complete loss of a carbon stock or sink pathway and dark red text represents the reduced efficiency of a pathway under warming. Sequestration pathways are in bold. Photograph of *Laminaria hyperborea* taken by Jonas Thomar. Organic carbon pathway diagrams are modified from [13]. Abbreviation: CO_2_, carbon dioxide.

When seaweed forests are lost or degraded, the carbon stored in their tissues is released into the water column in particulate or dissolved form. Because seaweed forests occur in shallow, well-mixed environments with rapid air–sea gas exchange, about half of this carbon can be released into the atmosphere (S1 Data). With the loss of seaweed forests also comes a reduction in the flow of dissolved and particulate organic carbon (POC) to ocean sinks. The majority of habitats that replace seaweed forests, such as turf algal reefs or sea urchin barrens [22,23], have either a smaller sequestration capacity or are emitters of carbon dioxide [24]. Seaweed forest loss or degradation will thus result in a net increase of dissolved carbon dioxide in surrounding waters and a net loss of sequestration capacity (Fig 1).

Warming can also have more subtle effects on seaweed carbon cycling. In regions where seaweeds are already reaching their temperature limits, warming can reduce carbon assimilation during seaweed growth [25]. Warming also increases the speed at which seaweeds are grazed, fragment, and release DOC [26], and accelerates how quickly this material is broken down [27]. This means that less organic carbon reaches deep, long-lived ocean sinks, resulting in a net increase of dissolved carbon dioxide in surrounding waters (Fig 1).

## Mismatch of scale between seaweed forest loss and successful conservation

In the following section, we first estimate reductions in carbon cycling benefits associated with ongoing global seaweed forest decline, then compare these losses to documented abatement gains from interventions from seaweed CDR.

### Climate-driven loss in kelp carbon sinks

Kelp forests, the most extensive and productive seaweed ecosystems on Earth [16], are listed by the Intergovernmental Panel on Climate Change (IPCC) as the second-most vulnerable marine ecosystem to climate change, after coral reefs [1]. Losses of kelp forests exceeding tens of thousands of hectares have been documented in Norway, Western Australia, and Japan [28,29]. Declines and localized extinctions have been reported across the North Atlantic ocean, New Zealand, Oman, and the northeastern Pacific ocean [30]. Overall, long-term records indicate that ~60% of kelp forests have declined over recent decades, while only ~5% have increased [31].

The best estimate of the global rate of kelp decline is a 1.8% loss per year in abundance, based on time-series data for these ecosystems [32]. Using a global area estimate of kelp forest extent of 641,224 km^2^ [33], this represents a lost area of 11,542 km^2^ of forest in 1 year. From a carbon emissions perspective, this loss in seaweed carbon cycling is equivalent to 5 million tonnes of carbon dioxide being released from the fraction of remineralized biomass that is not sequestered, 4 million tonnes of lost carbon dioxide in POC fluxes to deep ocean sinks, and 4 million tonnes of lost carbon dioxide in refractory DOC production annually, based on global estimates of per area NPP, POC export, and rDOC production [13–15]. If these rates of 1.8% instantaneous loss per year persist into the future, this is equivalent to 155 million tonnes of carbon dioxide in cumulative lost abatement from 2026 to 2030, and 2.5 billion tonnes of carbon dioxide by 2050 (S1 Data). These numbers are stark. However, the global extent of climate-driven loss remains uncertain due to large regions of the Earth being unmapped and limited long-term monitoring [32]. Using a more conservative prediction of 15.7% loss in global kelp extent by 2100 due to climate change, which is based on stacked species distribution models of kelp species [33] and does not consider changes in abundance but only areal change, the equivalent losses by 2050 are reduced by an order of magnitude. Yet, this rate of loss still represents 1.5 million tonnes of carbon dioxide lost annually, and 313 million tonnes of cumulative carbon dioxide abatement loss by 2050 (S1 Data). For a more concrete comparison that does not rely on global distribution models, if we consider kelp forest loss from six reported marine heatwave events over the past 25 years where the lost areas are well mapped [29,34–38], declines in kelp forests represent a total lost area of 97,438 ha and a cumulative loss of 8.2 million tonnes of carbon dioxide [39].

For some regions, we can also estimate how sublethal effects of climate change could reduce the effectiveness of these natural carbon sinks. Experiments of kelp carbon remineralization rates in the north Atlantic and northeast Pacific oceans suggest an average of a 9%–42% reduction in the potential of kelp carbon to be exported and sequestered long-term in deep ocean sinks as temperatures rise under different climate change scenarios (RCP4.5–RCP8.4) [27], although the RCP8.5 scenario is now unlikely. Based on the extent of kelp forests along these coastlines, which we estimate to be 206,235 km^2^ and have an overall kelp carbon drawdown of 94 million tonnes per year [15], this reduction is equivalent to a loss of 3.7–17.5 million tonnes of carbon dioxide not reaching deep ocean sinks every year (S1 Data). A similar decline in a seaweed carbon sink pathway can happen with lower productivity. In the United Kingdom, a 2.5 ℃ temperature increase resulted in a 33% loss of carbon drawdown from reduced growth rates in warm kelp forests compared to cool kelp forests [40]. In Western Australia, warming of 5 ℃ (from 15 ℃ to 20 ℃) is expected to cause a 37% decline in kelp carbon export due to less productivity and faster remineralization, which is equivalent to ~400,000 tonnes of carbon dioxide every year [41]. Temperature-driven changes to carbon sequestration through DOC pathways remain unresolved, which is an important knowledge gap given that >95% of seaweed carbon sequestration can occur though these pathways in some areas [21].

### Carbon abatement gains from kelp restoration

**Fig 2:**
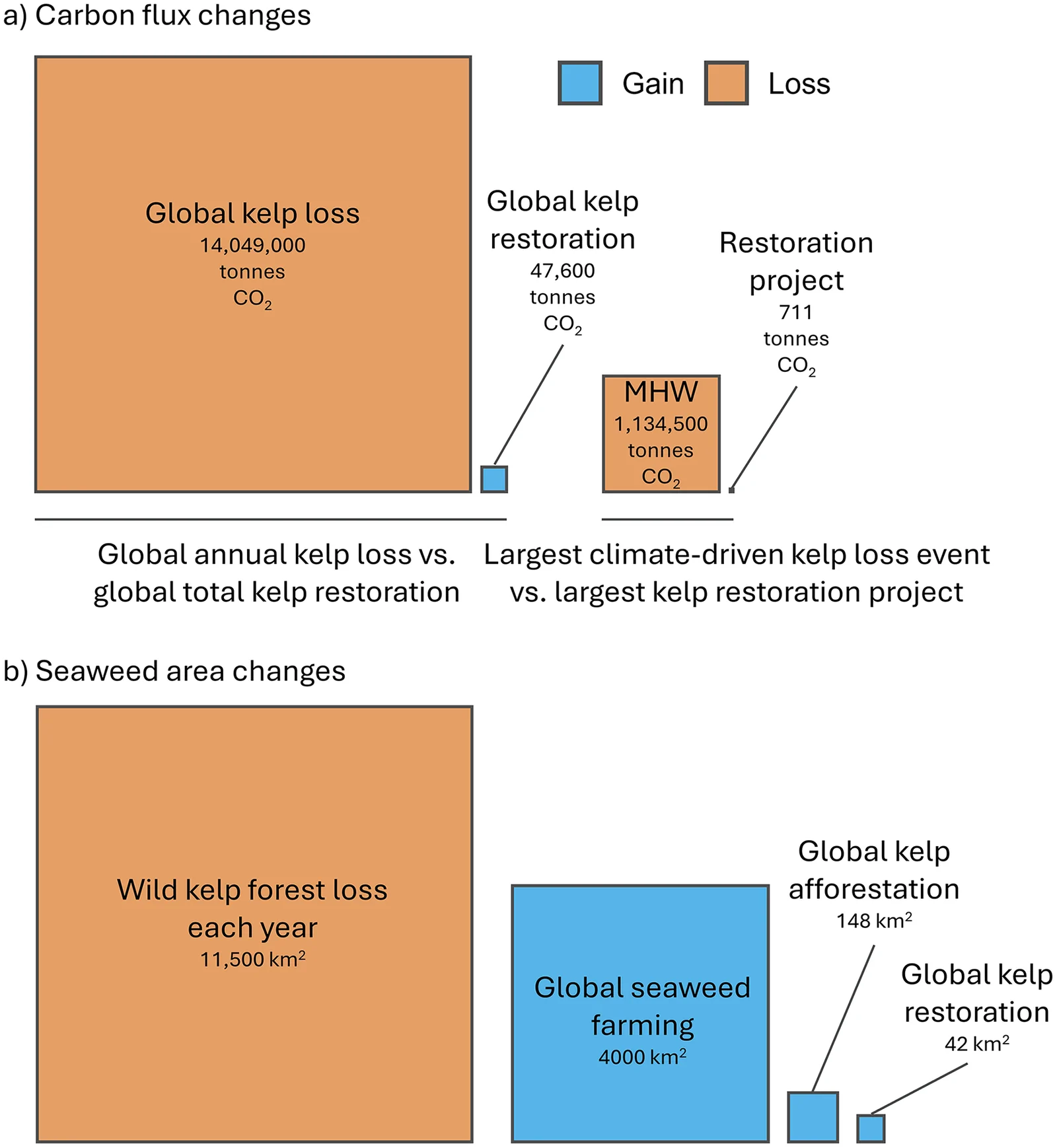
Mismatch in estimated carbon losses associated with ongoing kelp forest decline compared with documented kelp restoration gains. **a**) On the left side is a comparison of global estimates of annual kelp loss and total global kelp restoration. On the right side is a comparison of one year of carbon abatement loss immediately following the largest documented climate-driven kelp loss (2011 marine heatwave (MHW) in Western Australia [31]) to 1 year of possible carbon abatement gains from two of the largest documented natural kelp restoration projects globally: sea urchin removal restoration in northern Norway, and the Palos Verdes restoration project in California, USA [45,46]. **b**) Mismatch in estimated areas associated with ongoing annual kelp forest decline compared with current total global seaweed farming area (Laminariales, Fucales and red seaweeds) and area of documented kelp afforestation using artificial reefs and kelp forest restoration gains. Square areas are proportional to the reported magnitudes. Note that wild kelp forest area depicts an annual change in area compared to the total areal extent of seaweed farming. Abbreviation: CO_2_: carbon dioxide.

When we look at potential carbon abatement gains from kelp restoration, it is not keeping pace with these estimates of climate-driven loss (Fig 2). An optimistic estimate of the total wild kelp forest area successfully restored globally since 1958 is 4,200 ha (www.kelpforestalliance.org). If we assume these restored habitats immediately recover their ability to contribute to the carbon cycle, this represents an initial gain of 47,568 tonnes of carbon dioxide, followed by an annual gain of 27,936 tonnes of carbon dioxide each year (S1 Data). This is three orders of magnitude lower than our estimates of annual climate-driven loss. Based on area alone, we are predicted to lose 25 million ha of wild kelp forests by 2050, which is 6,108 times greater than the area we have restored in at least twice that time period. We did not include reported restoration areas from South Korea in this comparison (~14,686 ha of mostly afforested kelp forests) because these mainly involve adding artificial concrete reefs. Because of the intense emissions associated with concrete manufacturing, it could take a minimum of 4–13 years for these artificial habitats to offset the emissions associated with their own production, even without accounting for any of the emissions associated with transport and deployment of cement blocks [13]. However, we did include restoration areas from Japan (1,151 ha), even though an unknown fraction of these projects also involves artificial reefs. Furthermore, the reported gains from the restored areas we do include are also probably overestimates for several reasons. First, restoration takes time, and restored habitats may require several years before recovering their full carbon drawdown capacity [42]. Second, the long-term success of restoration projects is highly variable, and the persistence of restored kelp forest area is often uncertain because projects frequently lack the funding or capacity for long-term monitoring [43,44].

Successful kelp restoration projects reported to date have been much more limited in scale than kelp afforestation using artificial reefs, with the two largest verified kelp restorations being: 70 ha of kelp forest in northern Norway [46], which represents a CDR of ~710 tonnes of carbon dioxide and took 4 years of effort to remove sea urchins using quick lime; and 32 hectares of kelp forest in Santa Monica Bay, California, USA, which took 12 years of citizen science effort to eliminate ~6 million sea urchins [45] and represents a CDR of ~410 tonnes of carbon dioxide (based on average per area values of seaweed carbon cycling for these regions; S1 Data). We need 3,000–26,000 new projects like these each year to keep pace with ongoing potential carbon abatement losses from kelp degradation, 15,000 projects to draw down the carbon released by known marine heatwave events, and 7,000 projects to account for reduced future carbon cycling in the northern hemisphere alone under the most conservative warming scenario. Based on average costs for kelp restoration of 10,000–707,000 USD per ha [42], these interventions are equivalent to investing 974 million to 67 billion USD to restore areas known to be lost due to marine heatwaves alone, and 49 billion to 414 billion USD each year to keep pace with global rates of loss (based on median restoration costs for the two global rate of loss scenarios; S1 Data).

Overall, carbon release owing to climate-driven impacts on seaweed forests exceeds carbon drawdown through seaweed forest restoration by three to four orders of magnitude. This stark disparity highlights the need for a fundamental shift in how seaweed carbon is framed—away from an emphasis on future sequestration gains to an explicit assessment of carbon emission risks under accelerating climate change.

## When are interventions worth it from a CDR perspective?

Seaweed farming is another way that seaweeds are proposed to mitigate climate change [47,48]. Farming takes up dissolved carbon dioxide from the surrounding seawater and converts it to organic carbon, a portion of which can be buried under the seaweed farm or reach long-term carbon sinks and be effectively removed from exchange with the atmosphere [48]. Yet, seaweed farming only represents CDR if the farm activity results in greater greenhouse gas removal compared with what was naturally occurring in the area in which the farm was placed, which is not always the case. Farms in shallow areas can shade and damage seagrass meadows, whereas farms in open oceans may modify carbon removal by phytoplankton by ‘robbing’ them of limited nutrients [49]. On top of this, emissions associated with farming activities tend to also be large, on average 93 kg of carbon dioxide per ton of cultivated seaweed, which is far greater than additional carbon burial rates under seaweed farms (median burial values are equivalent to ~ 7 kg of carbon dioxide burial per ton of cultivated seaweed, based on yields of ~25 kg of carbon per ha) [50]. In a recent assessment of seaweed carbon burial rates and farm emissions, farm emissions entirely offset carbon burial in nine out of 11 farms where burial data were available, even when applying the lowest emissions reported in the literature for a given type of farmed seaweed [50]. This makes seaweed farming an unlikely method to mitigate climate change, although it is a promising way to sustainably produce low emissions materials and food. Finally, the scale of seaweed farming is small, at around 4,000 km^2^ [50], which is ~25% of the area of seaweeds lost to climate change each year (Fig 2b).

The narrative that seaweed forests are a natural climate solution and scalable CDR pathway comes with consequences for research and activities. For one, it puts a focus on areas that are already damaged. This is understandable, but ask an ecologist what is easier: preventing a kelp forest from being overgrazed by sea urchins or restoring an urchin barren that has already lost its kelp, and they will most likely reply that prevention is much easier [51]. For example, in Tasmania, Australia, experimentally increasing urchin predators in urchin barrens by establishing closed fishing areas and translocating ~1,000 large lobsters to each area had no impact on urchin densities and was not enough to recover the kelp forest in extensive barren areas after 12 years. By contrast, these same efforts were highly effective on remnant kelp forests with patchy barren areas that were showing early signs of overgrazing and had similar urchin densities to the extensive barren areas [52]. Similarly, for climate-driven collapse to turfs, kelp recruitment is inhibited by trapped sediment and altered chemical environments [53,54], making it much more difficult for kelp to regrow in these circumstances compared to in remnant forests that have not yet lost their canopy, even when that turf is removed [55].

**Fig 3:**
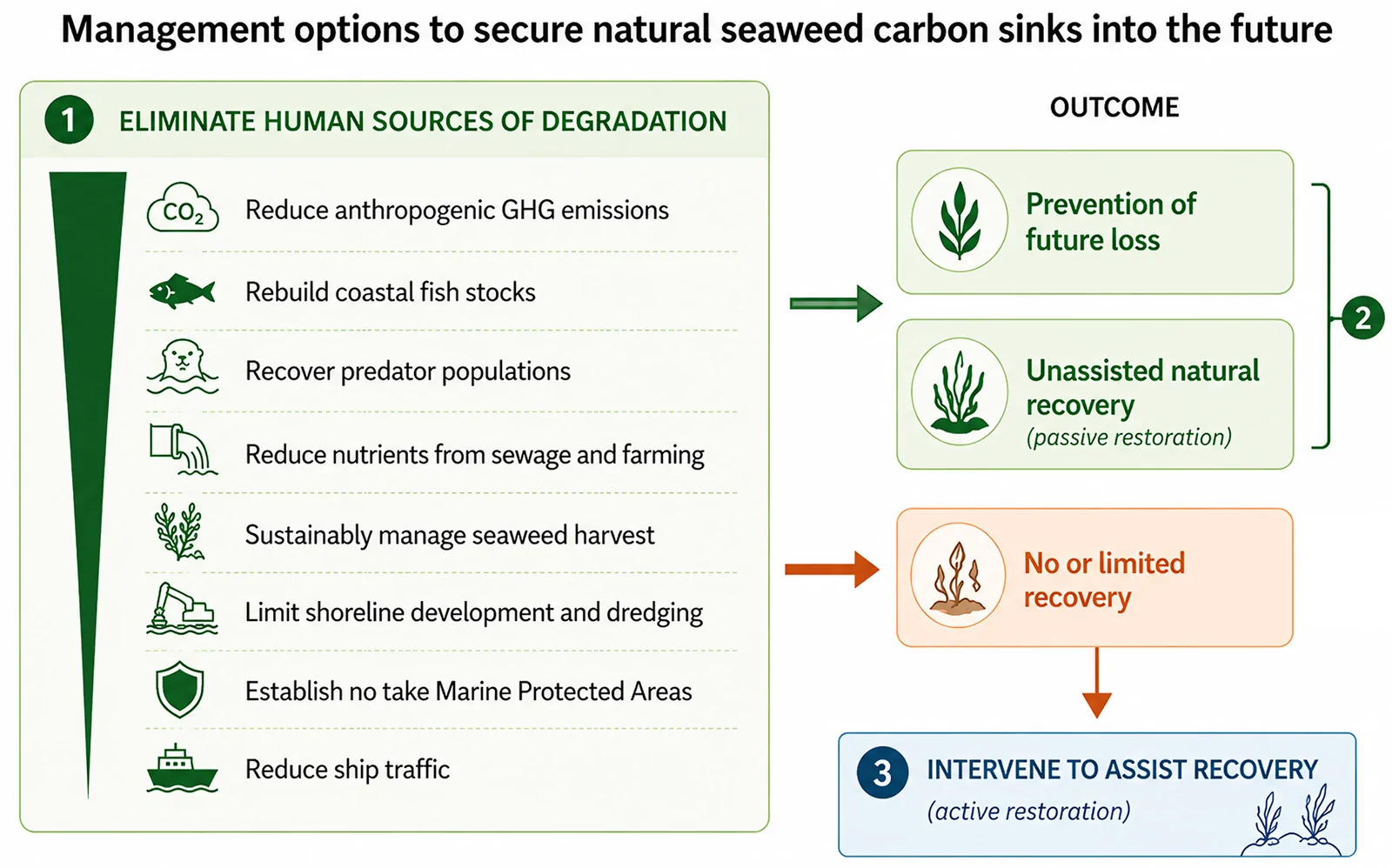
Interventions to reduce degradation of seaweed forests, listed in rough order of area affected by stressor. These actions (1) should be the first steps in seaweed forest conservation and can lead to prevention of future loss in intact areas or passive restoration in degraded areas (2). In cases where the outcome following step 1 is no recovery, additional active restoration interventions such as seeding, grazer control, or transplanting may be necessary (3). ChatGPT (OpenAI) was used to assist with figure presentation and graphical design [59]. Abbreviation: GHG, greenhouse gas.

When researchers study seaweed carbon cycling from a blue carbon or CDR perspective, they quickly run into the boundaries of IPCC accounting frameworks [56]. These frameworks tend to focus on the most certain carbon pathways and usually leave out others, such as DOC, where uncertainty is higher [12]. On top of that, CDR accounting hinges on additionality, which means that only carbon removal that happens because of a clear human action (such as restoration and protection interventions that increase seaweeds) can be counted, whereas protection against future loss and reduced pollution do not remove carbon dioxide, but instead represent avoided emissions. This sort of additionality works better for land forests, which are actively managed, replanted, and harvested at huge scales (although the quantification of CDR benefits for land forests remains somewhat contentious). Seaweed forests, by contrast, are mostly shaped by forces that are difficult to ‘directly control’ through local interventions or forces that are acting at large scales, such as climate-driven warming, centuries of overfishing of large fish stocks, and coastal darkening (Fig 3). Moreover, the misconception that natural ecosystems can effectively mitigate climate change carries the dangerous potential to misguide efforts to curb future warming and emissions. The carbon that marine ecosystems draw down and store on an annual basis remains constant if no habitat loss or changes to fluxes are occurring. Additionality depends on the additional removal of carbon through increased absorption, which can occur only if habitat area or rates of carbon drawdown increase as a result of human intervention. Protecting seaweed forests and preventing decline, therefore, can maintain the proportion of the carbon in the atmosphere that is absorbed by seaweeds on an annual basis and avoid further emissions through degradation; but does not necessarily draw down additional carbon. Natural ecosystems, including seaweed forests, can only truly act as a natural climate solution if restoration or protection results in increased habitat area and/or associated rates of carbon sequestration.

Restoration is costly and, in many areas targeted for restoration, the stressors responsible for the seaweed loss in the first place have not been fully mitigated, making successful outcomes difficult [44]. Restoration is also more costly than protection. Current costs for kelp restoration ranges from 10,000 to 707,000 USD per ha, whereas protection through managed and enforced marine protected areas ranges from 650 to 1,300 USD per ha [42]. In other words, we are placing our focus and funding on treating the most damaged and vulnerable areas where ongoing stressors make kelp recovery challenging and expensive, while vast tracts of seaweed forests that are currently drawing down carbon are not monitored or protected. Less than 2% of kelp forests are in fully protected areas [57]. Even so, marine protected areas are no silver bullet for kelp conservation, with ecosystems remaining vulnerable to climate change and major stressors on kelp requiring an additional suite of management solutions [58] (Fig 3). There is a clear need to better fund seaweed forest conservation and management to improve the efficiency, success, and scale of conservation actions. Ultimately, reducing greenhouse gas emissions remains a critical solution to prevent the continued loss of the ecosystem functions we already depend on.

There is also a risk that seaweed forest restoration becomes burdened with unrealistic climate expectations, mirroring what is happening to carbon offsets for terrestrial forests. Emerging evidence suggests that planning as though large-scale CDR will be feasible can delay fossil fuel phase-out and reduce near-term mitigation, leaving emissions higher by mid-century [60]. A rethink of seaweed blue carbon research away from CDR and towards preventing future climate-driven emissions pivots the focus towards urgently addressing climate change—not enabling further fossil fuel emissions through the hopeful prospect of future CDR (Box 1). The lessons from forests transitioning from sinks to sources are clear: we cannot assume that current natural carbon sinks will continue to function as our planet warms, and increasing natural carbon sinks cannot substitute for rapid emissions reductions.

**Box 1. Priorities for the field.**

**1. Resolve climate-driven changes in seaweed carbon sequestration**

This includes understanding:

- Rates of change in seaweed forest area and abundance
- Effects of warming on seaweed carbon cycling and fate
- Quantifying the carbon released during seaweed ecosystem collapse
- Carbon cycling functions of replacement ecosystem states (e.g., sea urchin barrens, turfs)

**2. Prioritize effective conservation of existing seaweed forests to avoid future loss rather than only repairing degraded areas**

Priority areas include understanding:

- Effects of area-based protection on seaweed forest resilience
- Effectiveness of reducing local stressors on seaweed performance (e.g., pollution, coastal darkening)
- Adaptive capacity and identifying climate refugia and resilient populations

**3. Address uncertainties around carbon sequestration by marine ecosystems**

- Improve estimates and verification methods around allochthonous carbon sequestration pathways
- Resolve dissolved organic and inorganic carbon pathways and fate, including refractory components

**4. Reframe seaweed conservation away from focusing on active interventions**

- Reframe seaweed restoration to include a suite of management options to reduce stressors, highlighting that active intervention is a final option
- Quantify other benefits from seaweed forests, including their role in supporting biodiversity, fisheries, improving water quality, and cultural identities and activities

## Conclusions

In this Essay, we still contend that seaweed restoration and farming are worthwhile. Reestablishing seaweed forests is an on-the-ground response to a global challenge and is critical to connect people with their local ecosystems. Even local kelp restoration success can provide important local and regional benefits for biodiversity and coastal function, as well as a range of other services [61]. Similarly, seaweed farming is a highly promising method to produce food and materials at low environmental costs, enabling us to reduce greenhouse gas emissions by replacing products with high carbon footprints with low-carbon seaweed products such as biodegradable plastics and biochar [50]. Our problem centers around the expectation that these activities can deliver meaningful climate change mitigation through CDR at the scales and speeds required to offset our growing emissions. Given the current pace of emissions and scale of climate-driven loss of seaweed forests, this expectation is not realistic.

The ocean is providing an essential service by absorbing a fraction of anthropogenic emissions [62]. If the world’s natural climate sinks start to become net sources, this natural buffer is lost, necessitating even steeper reductions in fossil fuel emissions to meet climate targets such as those set under the Paris Agreement [63]. The weakening and reversal of natural carbon sinks carries profound implications. If forests on land and below the water begin to act as carbon sources, climate change enters a more dangerous phase: one in which mitigation must race against both human emissions and destabilized natural climate feedbacks. It is not possible for us to meet our net-zero emissions targets without the help of these natural carbon sinks and, as they weaken, delays become more costly and partial solutions less effective. This pattern of carbon sinks transforming into carbon sources is already playing out on land and seems to also be happening in our more hidden marine forests. The most important question for seaweed blue carbon is no longer how much carbon these forests could sequester to mitigate climate change, but the extent to which they will continue to function as carbon sinks in a warming ocean.

## Supporting information

S1 Data Underlying data and calculations. (XLSX)

## Abbreviations

CDR: carbon dioxide removal
DOC: dissolved organic carbon
IPCC: Intergovernmental Panel on Climate Change
MHW: marine heatwave
NPP: net primary productivity
POC: particulate organic carbon
rDOC: refractory dissolved organic carbon.
